# Hyaluronan and Reactive Oxygen Species Signaling—Novel Cues from the Matrix?

**DOI:** 10.3390/antiox12040824

**Published:** 2023-03-28

**Authors:** Aikaterini Berdiaki, Monica Neagu, Ioanna Spyridaki, Andrey Kuskov, Serge Perez, Dragana Nikitovic

**Affiliations:** 1 Laboratory of Histology-Embryology, Department of Morphology, School of Medicine, University of Crete, 71003 Heraklion, Greece; 2 Department of Immunology, Victor Babes National Institute of Pathology, 050096 Bucharest, Romania; 3 Department of Technology of Chemical Pharmaceutical and Cosmetic Substances, D. Mendeleev University of Chemical Technology of Russia, 125047 Moscow, Russia; 4 Centre de Recherches sur les Macromolécules Végétales (CERMAV), CNRS, University Grenoble Alpes, 38041 Grenoble, France

**Keywords:** hyaluronan (HA), reactive oxygen species (ROS), reactive nitrogen species (RNS), tissue homeostasis, disease, ROS scavenging

## Abstract

Hyaluronan (HA) is a naturally occurring non-sulfated glycosaminoglycan (GAG) localized to the cell surface and the tissue extracellular matrix (ECM). It is composed of disaccharides containing glucuronic acid and N-acetylglucosamine, is synthesized by the HA synthase (HAS) enzymes and is degraded by hyaluronidase (HYAL) or reactive oxygen and nitrogen species (ROS/RNS) actions. HA is deposited as a high molecular weight (HMW) polymer and degraded to low molecular weight (LMW) fragments and oligosaccharides. HA affects biological functions by interacting with HA-binding proteins (hyaladherins). HMW HA is anti-inflammatory, immunosuppressive, and antiangiogenic, whereas LMW HA has pro-inflammatory, pro-angiogenetic, and oncogenic effects. ROS/RNS naturally degrade HMW HA, albeit at enhanced levels during tissue injury and inflammatory processes. Thus, the degradation of endothelial glycocalyx HA by increased ROS challenges vascular integrity and can initiate several disease progressions. Conversely, HA exerts a vital role in wound healing through ROS-mediated HA modifications, which affect the innate immune system. The normal turnover of HA protects against matrix rigidification. Insufficient turnover leads to increased tissue rigidity, leading to tissue dysfunction. Both endogenous and exogenous HMW HA have a scavenging capacity against ROS. The interactions of ROS/RNS with HA are more complex than presently perceived and present an important research topic.

## 1. Introduction

**Figure 1:**
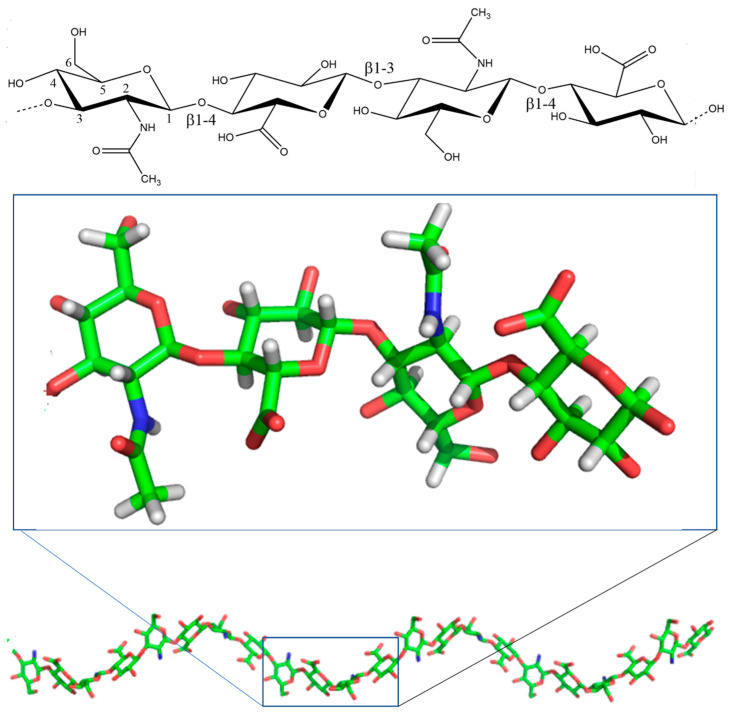
Chemical structure of two repeating units of HA; (β-D-GlcNAc 1→3 β-D-GlcA 1→4)n; 3-D representations of the low energy conformation of a tetrasaccharide unit, extracted from a longer segment shown in an extended two-fold helical conformation.

Hyaluronan (HA) is a naturally occurring non-sulfated glycosaminoglycan (GAG), cell-surface-associated biopolymer, and is the crucial component of tissue extracellular matrix (ECM). The chemical and structural characterization of GAGs is still a matter of intensive development [1]. HA is a regular polymer of disaccharides composed of glucuronic acid and N-acetylglucosamine linked via alternating β-1,4 and β-1,3 glycosidic bonds (Figure 1).

This GAG is widely distributed in vertebrate tissues and fluids, displaying remarkable physicochemical properties and biological effects. However, the occurrence of HA in tissues varies with, for instance, the following levels reported: human navel cords (4.10 mg/mL), human joint synovial fluid (1.50–3.60 mg/mL), vitreous humor (0.14–0.34 mg/g), brain (0.035–0.115 mg/g) [2], and human dermis and epidermis (0.20–0.50 and 0.10 mg/g, respectively). On average, HA turnover in vertebrate tissues equals 5 g per day and is provided by biosynthesis and enzymatic degradation [3]. Meanwhile, the turnover of HA in the blood flow reaches 30–100 mg daily.

## 2. HA Structure and Physicochemical Properties

### 2.1. Physicochemical Properties 

In healthy individuals, HA comprises about 10,000 (with variations) disaccharide units, which correspond to up to 40,000 kDa, and the chain can reach a contour length of several micrometers. HA is an elastoviscous component in synovial fluid that provides a smooth, gliding surface for joints to articulate. Such features are expressed in rheological properties, as HA exhibits a so-called Newtonian behavior at a low shear rate and a marked decrease in viscosity as the shear-rate increases. The HA chains tend to entangle at low concentrations, showing viscoelastic behavior, i.e., while they act as a viscous liquid at low frequencies, they show elastic behavior at a higher frequency. Other fundamental rheological properties (viscosity, static compression, elastic modulus in shear stress, and elastic modulus in compression) confer HA-based hydrogel with the properties that make it suitable for use as fillers in aesthetic medicine to shape the face or to treat the signs of facial aging. In the eye’s vitreous humour, HA supports and maintains a network of collagen fibers that protect the ocular tissues and maintain a clear visual path between the lens and retina. In solid tissues, the content of HA varies widely. Tissues such as cartilage and skin have extensive extracellular matrices in which HA binds and organizes proteoglycans, providing a highly hydrated network to resist tissue compression. Likewise, HA has a crucial role in the brain since this organ lacks the collagenous and elastic networks prevalent in other tissues. Indeed, HA and proteoglycan networks provide an alternative supportive matrix in brain tissue. A key role in the regulation of physiological functions as well as pathological processes has been attributed to HA deposited in the brain [4].

### 2.2. HA Turnover 

Three HA synthase enzymes (HAS1, HAS2, HAS3) localized in the plasma membrane synthesize and export high-molecular weight HA chains to the extracellular environment [5]. Newly synthesized HA chains in healthy tissue can be as large as 6000 kDa. At the cell surface, HA interacts noncovalently with the integral membrane receptor protein CD44 and other receptors through different binding modes that, in unison, define an interaction fingerprint. The conformational flexibility of HA chains facilitates such multivalent binding with proteins based on complementary surface densities in contrast to mere affinity. The multiplicity of interactions creates a complex peri-cellular matrix network that frequently undergoes further dynamic re-modeling and acts as a physiological protection of cells. HA has a high turnover rate at the cellular and tissue levels, mainly due to the enzymatic hydrolysis of hyaluronidases (HYALs), of which HYAL1 and HYAL2 are considered major HA-degrading enzymes in somatic tissue [6]. Indeed, HYAL2 localized to the cell surface is stabilized with a glycophoshatidyloinositol (GPI) anchor or is located in lysosomes, cleaving high molecular weight HA (HMW HA) to smaller, approximately 20 kDa fragments. Upon the internalization and transport of the fragments to lysosomes, further degradation to mainly tetrasaccharides is perpetrated by HYAL1 [6]. 

HMW HA is bound to receptor CD44 on the cell surface, often in the vicinity of HYAL2. HYAL2 can cleave HA chains of up to 20 kDa, corresponding to a chain of approximately 50 disaccharide residues. Such fragments are then internalized and transported to lysosomes. 

Mammalian hyaluronidase, HYAL1, is an endo-β-N-acetyl-hexosaminidases (EC 3.2.1.35; glycosyl hydrolase family) that hydrolyzes the β1→4 glycosidic bond of HA into various oligosaccharide lengths, the shortest of which are tetrasaccharides. For stereochemical reasons, the β1→3 glycosidic bond is resistant to enzymic cleavage. The crystal structure reveals a molecule composed of two closely associated domains:  a catalytic domain that adopts a distorted [β/α]8 barrel resembling that of bee venom hyaluronidase and a novel, EGF-like domain, characteristic of involvement in protein−protein interactions and regulatory processes [7]. By catalyzing the hydrolysis of hyaluronan, hyaluronidase lowers the viscosity of hyaluronan, thereby increasing tissue permeability.

Recently, HYBID (hyaluronan binding protein involved in hyaluronan depolymerization), alias KIAA1199/CEMIP, has also been shown to depolymerize HA into small- and intermediate-sized fragments, thereby contributing to HA degradation [8]. Indeed, as discussed by Spataro et al., HYBID depolymerizes HA that has been internalized via clathrin-coated vesicles into early endosomes. Subsequently, the degraded HA fragments are excreted into the extracellular space [9].

### 2.3. ECM Organization

In native tissues, the noncovalently assembled HA network interacts with another GAG: chondroitin sulfate, which is incorporated into proteoglycans such as versican, neurocan and aggrecan [10]. The HA-based matrix further acts as a protective target for reactive oxygen and nitrogen species (ROS/RNS) generated during inflammation and limits penetration of these species to the cell membrane [11]. However, excessive cleavage/degradation of HA and release of bioactive fragments facilitate pathologies, including inflammation and cancer [12]. Effects other than the ECM organization, such as immunomodulation and various cellular processes, occur. Environmental cues such as tissue injury or infection change downstream signaling functionalities of HA. 

HA is susceptible to degradation by hydroxyl radicals, peroxynitrite, and hypochlorite anion, all of which can be created in vivo and are increased during inflammation [13]. Unlike native HA, such fragments of HA have diversified effects on inflammation, cancer, fibrosis, angiogenesis, and the autoimmune response. Inflammation is associated with a potential reduction in HA molecular mass and the resulting production of bioactive “danger signal” fragments throughout cell signaling pathways which depend on the status of the HA component and the peri-cellular matrix [14]. The degradation of HA results in the presence of low molecular weight (LMW) HA fragments, which disrupt the clustering of CD44 receptors at the cell surface while increasing the availability for interactions with other receptor proteins such as TLR4, TLR2, and RHAMM [15].

### 2.4. Mechanisms for HA Degradation by Reactive Oxygen Species (ROS)

In vivo, degradation of HA may arise from hydroxyl radicals, peroxynitrite, and hypochlorite anion and is likely to be increased in the context of inflammation. There exists a rich literature describing the mechanisms leading to the production of reactive oxygen species (ROS) or reactive nitrogen species (RNS) in biological systems [13], as well as attempts at mathematical and computational modeling [16]. ROS/RNS can be classified as either free radicals containing one unpaired electron or nonradicals. The former generally includes superoxide radical (O_2_^•−^), hydroxyl radical (^●^OH), NO radical (^●^NO). peroxyl (ROO^●^), and alkoxyl radicals (RO^●^). The latter contains hydrogen peroxide (H_2_O_2_), hypochlorous acid (HClO), peroxynitrite (ONOO^−^), ozone (O_3_), singlet oxygen (1O_2_), aldehydes, and organic peroxides [17]. 

Superoxide anion radical (O_2_^•−^): The reduction of the oxygen molecule is the reaction by which animal cells produce metabolic energy (O_2_ + 4e^−^ + 4H^+^ = 2H_2_O). Along this reaction, several sub-cellular structures reduce O_2_ molecules producing the superoxide anion radical O_2_^•−^, which in an aqueous (acidic) milieu forms per hydroxyl radical ^●^O_2_H. O_2_^•−^is formed in neutrophils, monocytes, macrophages, and eosinophils due to the action of NADPH oxidase enzyme, also called respiratory burst oxidase. NADPH oxidase, a highly regulated enzyme complex composed of several proteins, reduces oxygen to a superoxide anion radical at the expense of NADPH [18]. Hydroxyl Radical ^●^OH, the hydroxyl radical, is the most reactive ROS. The degradation of HA by hydroxyl radicals and its protection by free radical scavengers are well documented. The source of free radicals in biological systems is termed the Fenton reaction, which implies the participation of a transition metal cation (e.g., Fe^2+^): 

[(Fe^2+^ + H_2_O_2_ → Fe^3+^ + OH^−^ + ^●^OH]

In this reaction, Fe^3+^ can be reduced back to Fe^2+^ [19]. 

**Figure 2:**
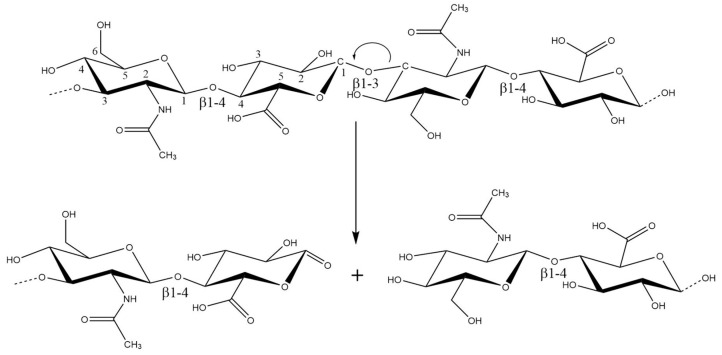
The radicals at carbons, which form glycosidic bonds, undergo a β-scission reaction resulting in the breakdown of the HA chains at C1 of the GlcA residue.

The hydroxyl radical can abstract hydrogen atoms at all ring C-H bonds except C-2 of N-acetyl hexosamine. The abstraction of hydrogen atoms generates carbon-center radicals. The radicals at carbons, which form glycosidic bonds, undergo a β-scission reaction resulting in the breakdown of the HA chains [20] (Figure 2). Under physiological (healthy) conditions, the iron ions are always firmly bound. In blood, they circulate associated with the protein transferrin, and in cells, they are stored and linked to the protein ferritin. Nevertheless, cells observe an increase in the so-called “labile iron pool” under stress conditions. The source of superoxide anion in biological systems are activated PMNs (polymorphonuclear leukocytes). 

Nitrogen Monoxide (^●^NO): The bioactive free radical nitrogen monoxide (^●^NO) is produced in various cells/tissues by the enzyme NO synthase. O_2_^•−^and ^●^NO are the precursors of different reactive oxygen species (ROS), including hydrogen peroxide, peroxynitrite, and hypochlorous acid. 

Hydroxyl Peroxide: H_2_O_2_ is produced from two superoxide anion radical species (O_2_^•−^+ O_2_^•−^+ 2H^+^ → H_2_O_2_ + O_2_). In vivo, the family of super oxide dismutases (SOD) catalyze such a reaction (the cytosolic SOD form contains Cu and Zn, and the mitochondrial form contains Mn). 

Hypochlorous acid (HOCl) and Hypochorite (OCl^−^): Myeloperoxidase enzymes of PMNs can generate hypochlorite ions (H_2_O_2_ + Cl^−^OCl^−^ + H_2_O). Methionine blocks this reaction. Hypochlorite anions may function by participating in the production of hydroxyl radicals. N-acetyl residues react with hypochlorite to generate polymer-derived N-chloro derivatives. The decomposition of these derivatives gives rise to nitrogen-centered radicals. They undergo rapid intramolecular abstraction reactions to give carbon-centered radicals at C-2 on the amino sugar rings via a 1,2-hydrogen atom shift [21] and/or at C-4 on the neighboring glycosidic residues (via 1,5-hydrogen atom shifts) [22]. These products cleave glycosidic bonds through β-scission [21]. 

Peroxynitrite (ONOO^−^): Peroxynitrite can be generated by the reaction of nitric oxide, produced by the nitric oxide synthase enzymes (NOS), with superoxide anion, released by activated polymorphonuclear leukocytes (PMN) (O_2_^•−^+ ^●^NO → ONOO^−^), which yields the formation of two radicals: (ONOO^−^ + H^+^ → ONOOH → ONO^−^ + ^●^OH). An increased expression of NOS is common in inflammatory disease processes. Peroxynitrite degrades HA via a hydroxyl radical-like mechanism [23].

ROS-generated HA fragments may not have the same biological effects as those created by enzymatic cleavage. Therefore, several factors must be considered to characterize ROS-induced alterations to GAGs in vivo [17,24]. The first factor is the GAGs’ proximity to the adjacent ROS/RNS production sites as these highly reactive species have a relatively short life span. Furthermore, the concentration and levels of antioxidation need to be sufficiently high and low, respectively. ROS-mediated HA degradation occurs randomly and generates products with a polydisperse size. Not only are glycosidic linkages cleaved, but there is the possibility of other structural changes, including ring opening and the occurrence of different conformational characteristics [25], which would affect their downstream reactions. As a result of their depolymerization effects, ROS/RNS could serve as the substrates for the following enzymatic cleavage, thereby facilitating further HA degradation. One possible explanation is that the partial degradation of HA would overcome steric factors which prevent the interaction between the hydrolases and their highly polymerized substrates.

## 3. Mechanisms of HA Action 

The net effect of parallel HA synthesis and degradation determine the amount of HA and its molecular weight at a given time and tissue location. Consequently, the produced HA will display its biological effects depending on its molecular weight [26,27]. 

HA functions are mostly perpetrated through interactions with HA-binding proteins (hyaladherins) localized to the cell membranes and the ECM. HA anchors to the cells by binding to its cell surface receptors, including CD44, RHAMM, HA receptor for endocytosis/stabillin-2 (HARE), lymphatic vessel endothelial HA receptor-1 (LYVE-1), TLR2, and TLR4, as reviewed by Roedig et al. [28]. It is important to note that HA is an unsulfated GAG and, thus, not charged. Therefore, depending on its molecular weight and individual receptor expression, downstream signaling pathways are activated upon HA binding, modulating cell growth, migration, inflammatory activities, and cell differentiation [27]. The effect of HA is perpetrated in both an autocrine and paracrine manner [29]. Moreover, due to its physicochemical properties, HA can act as a structural molecule affecting tissue hydration, ECM organization, and osmotic balance [30].

### 3.1. CD44

CD44 is the main HA cell membrane receptor. Even though a single gene encodes CD44, a standard form (CD44s) and different isoforms have been identified (v1–v11) due to alternative splicing [31] and post-translational modifications, including N- and O-glycosylation and glycosaminoglycanation [32]. CD44s, the most abundant form, exhibits the prototype structure consisting of a distal extracellular domain (bearing the ligand-binding sites), a single-pass transmembrane domain, and a cytoplasmic domain [31]. The crystal structure of the extracellular domain of C44 has been resolved [32]. Thus, the extracellular domain, carrying a link module with a highly conserved α/β fold flanked by two conserved disulfide bridges [33], binds HA in a manner defined by hydrogen-bonding and shape [32]. Furthermore, extensions of the module with specific sequences have been determined. Therefore, this enlarged binding domain can be modified, justifying the effect of receptor modification on HA binding. An interaction fingerprint of the respective CD44-binding, consisting of different binding models, has been revealed through computational modeling [34]. Binding induces conformational changes conveyed to the intracellular domain and regulates CD44 aggregation on the cell membrane [32,34]. Indeed, HMW HA stimulates CD44 clustering, whereas LMW HA inhibits it [35]. Notably, as discussed by Noar et al., in some cases, to bind HA, CD44 needs to be stimulated, activated or deglycosylated [36]. Indeed, it seems that CD44 N-glycosylation controls the “on” or “off” switch of HA binding to CD44 [37,38]. 

Upon activation, the intracellular CD44 domain binds with the cytoskeletal linker protein ankyrin and the ERM linkers, denominated as ezrin, radixin, and moesin (ERM) [39,40]. By engaging cytoskeletal linker proteins and respective downstream signaling pathways, CD44 can regulate most biological functions, including growth, migration or adhesion [40,41,42,43]. For example, the interaction of HA with CD44 regulates receptor-mediated HA internalization cell aggregation, adhesion, migration, and proliferation [44]. 

The binding of LMW HA and HMW HA with CD44 differs and exerts discrete effects. Thus, LMW HA induces pro-inflammatory and pro-angiogenic effects facilitating cell growth and motility [45], whereas HMW HA exerts anti-angiogenic and anti-inflammatory effects [46]. For this reason, LMW HA has been characterized as a danger-associated molecular pattern (DAMP) [28,47,48]. Furthermore, upon binding of LMW HA to CD44 Src Kinase, focal adhesion kinase (FAK) and ERK1/2 are phosphorylated to stimulate cell growth, motility, and angiogenesis [49].

However, some studies suggest that the oxidation of CD44 and HA decreases their mutual binding affinity, probably attenuating tumor cell growth and necrosis [50]. Therefore, targeted modulation of CD44/HA correlated to cancer growth inhibition could be explored as a novel direction in antitumor therapy. 

### 3.2. RHAMM

The receptor for HA-mediated motility (RHAMM) was determined initially to be a soluble protein exhibiting HA-binding properties [51]. Various isoforms of RHAMM exhibiting discrete MWs have been localized to the cell surface and the cytoplasm/nucleus [52]. RHAMM isoforms result from alternative splicing, exhibit discrete localization and expression correlated to the respective cell type and status [53,54] and also have effects on HA regulation [55,56]. 

RHAMM protein does not carry a transmembrane domain, making its interaction with other cell membrane receptors obligatory to regulate downstream signaling. For example, CD44, transforming growth factor β1 (TGF-β1), and platelet-derived growth factor (PDGFR) have been identified as co-receptors [57]. Notably, the interactions of HA with RHAMM/HA regulate cell growth and migration [54,58,59,60]. Indeed, as discussed by Garantziotis and Savani, 2019, when HA binds to membrane RHAMM, a transient burst of protein tyrosine phosphorylation is elicited, followed by increased focal adhesion turnover and Ras/ERK1/2 regulation [54]. Engagement of HMW HA with membrane RHAMM initiates PI3K-dependent Rac activation and increased migration of arterial smooth muscle cells [61]. LMW HA stimulates RHAMMs’ intracellular expression, co-localization with ERK1/2 [53] and β-catenin [61] to facilitate fibrosarcoma cell migration and growth [62].

### 3.3. HARE

HA receptor for endocytosis (HARE) is expressed by various endothelial cells, including those of the liver, lymph nodes, bone marrow, and spleen, as well as in the specialized structures of the brain, eye, heart, and kidney in mice [63]. HARE is a proteolytic isoform of Stabilin-2 that binds explicitly and internalizes many ligands, including HA, which leads to their degradation [5]. As such, HARE executes the systemic clearance of HA from the vascular and lymphatic systems utilizing coated pit-mediated uptake. Furthermore, HARE exhibits intracellular localization, mainly within the endocytic system, suggesting that it is transiently present on the cell surface, which is characteristic of active endocytic recycling receptors. Interestingly, HARE binding HA fragments of 40–400-kDa initiates NF-κB-activated gene expression, suggesting that this receptor can detect a specific size range of HA degradation products correlated with pro-inflammatory signaling [64].

### 3.4. LYVE-1

LYVE-1, a transmembrane glycoprotein, is a significant HA receptor expressed by the lymphatic endothelial cells. LYVE-1 binds HA and is involved in its clearance. However, the binding requires specific receptor self-association and HA aggregation [65]. This HA receptor is predominantly localized in the overlapping junctions of lymphatic capillaries serving as entry points for migrating inflammatory cells. Indeed, engaging HA incorporated into antigen-presenting dendritic cells’ glycocalyx facilitates these cells’ docking and transit [65]. Moreover, the ability of dendritic cells and macrophages to adhere to the lymphatic endothelium and subsequently transmigrate through it highly depends on LYVE-1 clustering, which is triggered by selective interactions with the HMW glycocalyx HA [58]. LYVE is also obligatory for T-lymphocyte antigen-specific responses [66]. A recent study demonstrated that resident macrophages expressing LYVE-1 interact with the pericellular HA matrix of smooth muscle cells, enhancing matrix metalloproteinase MMP-9-dependent proteolysis. The increased collagen proteolysis attenuated its deposition and inhibited arterial stiffness in a mouse model [67]. 

### 3.5. TLR2 & 4

TLRs are critical receptors the innate immune system utilizes to identify microbes and subsequently trigger the process of the host defense [68]. As previously discussed, TLR4 has been established as the transmembrane signaling protein needed for the LPS-dependent downstream signaling in macrophages, whereas TLR2 mediates the recognition of mycobacteria and gram-positive bacteria by macrophages [69].

**Figure 3:**
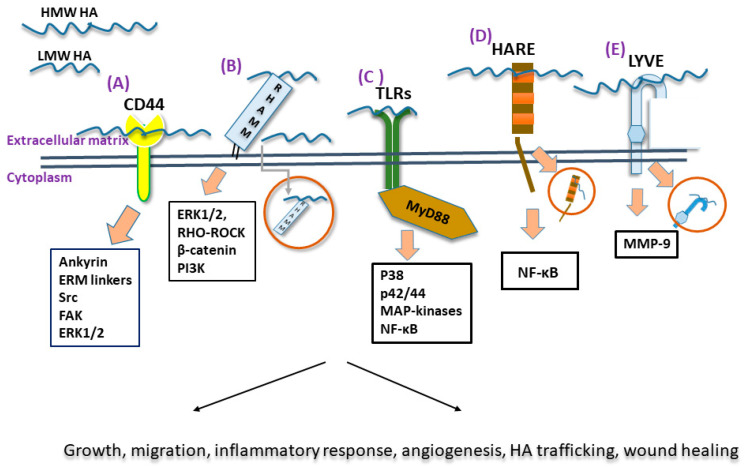
Mechanisms of HA action. (**A**) Upon activation, the intracellular CD44 domain binds with the cytoskeletal linker protein ankyrin and the ERM linkers, denominated as ezrin, radixin, and moesin (ERM), to regulate cell functions. Upon binding of LMW HA to CD44 Src Kinase, FAK and ERK1/2 are phosphorylated to stimulate cell growth, motility, and angiogenesis. (**B**) The binding of HMW HA to RHAMM regulates Ras/ERK/12 activities and initiates PI3K-dependent Rac activation and increased migration of arterial smooth muscle cells. (**C**) The binding of LMW HA to TLRs induces MyD88, downstream MAP-kinase activation and Nf-κβ nuclear translocation to enhance the inflammatory response. (**D**) HARE executes the systemic clearance of HA. In contrast, LMW HA binding initiates NF-κB-dependent gene expression. (**E**) LYVE-1 is involved in HA clearance and endothelial transmigration of lymphocytes. An LYVE-1-dependent interaction of macrophages with the pericellular HA matrix of smooth muscle cells enhances MMP-9-dependent inhibition of arterial stiffness.

It has previously been discussed that lower molecular mass forms of HA (<500 kDa), especially in the 100–250 kD range, activate inflammatory cells by binding to TLR2 and TLR4 [69]. Indeed, the cleavage of HMW HA into LMW HA, which is limited in homeostatic tissues, is enhanced in injury, inflammation, and re-modeling events; it is a part of the initial tissue response for maintaining homeostasis [70]. Upon binding of LMW HA to TLR4, nuclear translocation of nuclear factor (NF)-kappa B perpetrated by MAP-kinases is determined in dendritic cells (DCs), signaling these cells’ inflammatory activation [71]. The interaction of HA with its receptors and downstream mechanisms are summarized in Figure 3.

## 4. HA-Associated ROS Effects in Tissue Homeostasis 

The abundance and MW of HA are vital in maintaining tissue homeostasis. ROS, including hydrogen peroxide, superoxide, peroxynitrite, nitric oxide, and hypohalous acids, naturally degrade HMW HA in the organism, albeit at enhanced rates during tissue injury and inflammatory processes [72]. Vascular integrity, obligatory to maintaining tissue homeostasis, is partly regulated by the endothelial glycocalyx and cell–cell junctions. Defects in endothelial barrier function are an initiating factor in several disease processes, including atherosclerosis, ischemia/reperfusion, tumor angiogenesis, cancer metastasis, diabetes, sepsis, and acute lung injury.

HA is an essential component, accounting for up to 20% of the endothelial glycocalyx (EG), a gel-like layer covering the endothelial cell luminal surface [73]. In addition to HA, glycocalyx consists of membrane proteoglycans, glycoproteins, and adhered plasma proteins [74]. The EG is crucial in vascular homeostasis as it modulates the microvascular tone and vascular permeability, regulates leukocyte adhesion on the endothelium, and abrogates microvascular thrombosis [75]. Indeed, the impairment of EG is an established early step in initiating chronic vascular complications [12], such as those presenting in atherosclerosis, diabetes, ischemia/reperfusion, cancer metastasis, acute lung injury, and sepsis. 

Two layers of EG have been defined. The outer layer consists mainly of GAGs and plasma proteins, which regulate and prevent excessive blood cell attachment to the endothelium [73]. HA and PGs are abundant in the inner layer handling key endothelium roles. The abundance of HA in the inner layer of the EG contributes to vascular homeostasis. Under disease conditions, e.g., community-acquired pneumonia (CAP) and acute respiratory distress syndrome (ARDS), significant HA shedding correlated with ROS production, and not with changes in the expression of HYALs, is evident [76]. Notably, in ARDS, HA shedding is associated with inflammation, endothelial cell activation status as well as the disintegration of the basement membrane [76]. An accumulation of HMW-sheared HA was determined in the plasma of the most severe ARDS and CAP patients. Such an accumulation could signal a counter-regulatory mechanism triggered to attenuate inflammation. However, it could also indicate “raw” EG shedding in response to high ROS production and augmented inflammatory reactions [73].

It has been established that glycocalyx permeability mainly depends on the EG’s HA and chondroitin sulfate content [11]. Moreover, HA is found to be the critical factor able to transduce shear stress regulating endothelial NO production and vessel tone, as earlier discussed [77]. Likewise, HMW HA enhances vascular integrity through caveolin-1-dependent mechanisms [78]. Thus, endothelial barrier disruption in the lung results in the leakage of proteins, fluids, and inflammatory cells into alveoli, leading to pulmonary edema.

HMW-HA was shown to activate CD44s signaling and facilitate lung endothelial barrier integrity through S1P1/Rac1 downstream signaling, resulting in cortical actin formation. On the other hand, LMW HA release, partly mediated by ROS, triggers CD44v10 signaling and barrier damage through an S1P3-dependent axis [79]. 

As an essential component of the ECM, HA is a critical player in regulating tissue stiffness. Thus, the normal turnover of matrix components, including HA, protects against matrix rigidification and attenuation of tissue plasticity due to excessive cross-linking, which could partly be initiated by free radicals’ action [5,80]. Conversely, insufficient turnover would lead to the creation of gels exhibiting increasing rigidity, ultimately leading to tissue dysfunction, impairment of physical movement, and inadequate infiltration of immune cells [5].

HA participates in skin homeostasis and is implicated in keratinocyte differentiation, as recently discussed [14]. Interestingly, it has been shown that CD44 maintains the important skin barrier function, the pericellular keratinocyte matrix [81]. HA, likewise, exerts a vital role in wound healing. Indeed, ROS-mediated HA modifications can facilitate or attenuate TLR downstream signaling to modulate the status of the innate immune system essential to tissue regeneration [82]. Specifically, HA participates in the homeostasis and inflammation phases of wound healing that are characterized by increased HA production and rapid subsequent degradation due to HYAL and generated ROS actions. The released LMW HA facilitates HAS genes’ transcription, followed by increased HA synthesis and a generation of a feedback loop enhancing the inflammatory response and the first phase of wound healing [83]. The maximum deposition of HA is initially achieved, with a concomitant downregulation, upon infiltrating the proliferating fibroblasts. The fibroblasts, in turn, produce proteoglycans needed for the polymerization of collagen obligatory for wound closure [83].

HA is also suggested to protect against ROS-induced DNA damage. Indeed, HARE’s uptake of HMW HA in WI-38 fibroblasts attenuated DNA damage induced by both exogenous and endogenous oxidants [84]. Likewise, HA is postulated to attenuate ROS effects in mesenchymal stem cells [85].

These studies convincingly show the critical role of ROS-correlated HA effects in maintaining tissue homeostasis obligatory to the physiological functioning of all organs. 

## 5. ROS-Generated HA in Inflammation 

### 5.1. Inflammation and HA 

Inflammation is still a subject of intense study [86], as acute and chronic inflammation are sustained by various mechanisms that are still not fully understood. Cell–matrix interactions regulate cellular homeostasis, and any alteration in ECM integrity promotes the initiation and progression of inflammatory-related diseases [87]. HA fragments of various molecular sizes as endogenous ECM molecules, partly produced through ROS action, induce discrete biological effects. Thus, in inflammatory-related pathologies, highly polymerized HA is transformed into smaller molecules that increase the inflammatory response by activating supplementary inflammatory factors, such as ROS production and inflammatory immune molecules such as cytokines expression, or by enhancing the production of chemokines and other enzymes that recruit additional leukocytes [88]. 

At the same time, LMW HA degradation products can induce inflammation [86] partly mediated through Toll-like receptors (TLRs). A recent study showed that HA tetra-saccharide is the smallest fragment to augment inflammation using HA oligosaccharides of different molecular weights and modifications [89]. On the other hand, the HA disaccharide (ΔHA2), which bears an unsaturated double bond at the non-reducing end, was proven to block TLR4-dependent inflammation competitively. Deacetylated HA disaccharide (dHA2) and chondroitin disaccharide (CH2) did not attenuate TLR4-induced gene expression. These authors conclude that anti-inflammatory activity strongly depends on the HAs’ polymerization degree, acetylation degree, and actual configuration. The study also underlined that HA disaccharide reduced in vivo pro-inflammatory cytokines production, suggesting its therapeutical anti-inflammatory potential [90]. 

Conversely, larger HA oligosaccharides can activate TLR4 on human dendritic cells (DC) and induce their functional maturation. The earlier study of Tremeer et al. showed that phosphorylation of p38/p42/44 MAP-kinases and nuclear translocation of nuclear factor (NF)-kappa B occur in DCs, proving the TLR-4 signaling pathway activation [71]. Other studies have shown that LMW HA fragments increased cytokine production. Indeed, a recent elegant study demonstrated that LMW HA (15–40 kDa) isolated from obese patients induced in normal PBMC a significant increase in the expression of the pro-inflammatory cytokines IL-1β, MCP-1, and IL-8. This study reinforced the pro-inflammatory action of LMW HA and emphasized the involvement of small HA fragments in metabolic deregulations [90]. In bladder cancer, the increased fragmentation of HA and the increased LMW HA deposition in the tumor site are associated with enhanced production of multiple inflammatory cytokines, chemokines, and angiogenic factors [91]. 

HA binds on the immune cells via CD44, which regulates Hyal2 potent HA-degrading activity. In addition, a recent study has shown that when Hyal2 is activated, IL1β secretion is triggered, enhancing the pro-inflammatory milieu. Thus, within the tumor, the myeloid cells expressing Hyal2 sustain LMW HA accumulation and tumor-associated inflammation [90]. The increased expression of the HA-degrading protein HYBID has been correlated to inflammation, hyperplasia, and fibrosis in human synovial membrane biopsies taken from osteoarthritis patients [92].

An interesting earlier study has shown that macrophages isolated from CD44 null mice can be activated with LMW HA, proving that TLRs are involved in HA-induced intracellular signaling [93]. Indeed, HA is involved in the immune response by activating or inhibiting TLR-mediated intracellular signaling pathways, activating the first line of innate immune cells. 

Adaptive immune cells can also be regulated through their HA receptors [94]. Thus, T lymphocytes exhibit increased CD44-dependent HA binding when subjected to antigen activation, favoring lymphocyte infiltration into the inflammatory site [95]. On the other hand, HMW HA enhances Foxp3 expression in the Treg subpopulation, which induces IL-2, IL-10, and TGF-β production and dampens inflammation [96]. Under inflammatory conditions, dendritic epidermal T cells (DETCs) secrete keratinocyte growth factors 1 and 2 that stimulate skin cells’ HA production and enhance macrophage recruitment [96]. 

Not only are T cells “sensitive” to HA, but B lymphocytes are as well. In murine models of inflammation, HA application induces B cells chemotaxis to the inflammatory site, where the cells increase their IL-6 and TGF-β production [97]. Later reports by Iwata et al. have proven that LMW HA stimulates B cells via the TLR4 receptor and endows these lymphocytes with increased secretion of IL-6 and TGF-beta [97]. In addition, LMW HA promotes CD44 association with several other receptors (TLR2, TLR4), downregulating CD44 association that would lead to NF-κB-specific transcriptional activation. This well-known activation enhances pro-inflammatory cytokines, IL-1β, and IL-8 expression, even in tumor cells, including human breast cells [98].

Comprehensive studies have shown that LMW HA induces inflammation activating TLR receptors and initiates the production of pro-inflammatory cytokines/chemokines. This LMW HA role can also be beneficial. In acute inflammation, e.g., in wound healing, the activation of immune cells is critical. Therefore, in this milieu, the LMW HA fragments would augment the activation of the wound-healing process. On the other hand, maintaining a constant inflammatory microenvironment in chronic wounds can impede the healing process [99]. 

Contrary to LMW HA, HMW HA has anti-inflammatory and immunosuppressive effects. In the most common form of arthritis, osteoarthritis, this anti-inflammatory effect is already clinically documented. Therefore, recent extended studies have shown that intra-articular injection of HMW HA has long-term effects in improving the condition compared with other intra-articular treatments [100]. Recent reports have shown that platelet-rich plasma combined with HMW HA favored joint repair [101]. On a molecular level, earlier studies performed in animal models of osteoarthritis have shown that HMW HA inhibits IL-1β expression in synoviocytes [102]. This finding was later confirmed in human fibroblast-like synoviocytes from osteoarthritis patients [103]. Furthermore, if CD44 is blocked, the HMW HA effect on pro-inflammatory cytokine gene expression is reduced [98], a finding that emphasizes the importance of the CD44 signaling pathway in the HMW HA functions. In an animal model of osteoarthritis, HMW HA significantly reduced TLR4, TLR2, MyD88, and NF-kB expression in synoviocytes along with reduced mRNA and protein production for several pro-inflammatory molecules (e.g., TNFα, IL-1β, IL-17, MMP-13, inducible nitrous oxide synthase). These findings highlight that in this animal model, HMW HA can reduce the pro-inflammatory process in the early inflammatory phase of osteoarthritis. 

**Figure 4:**
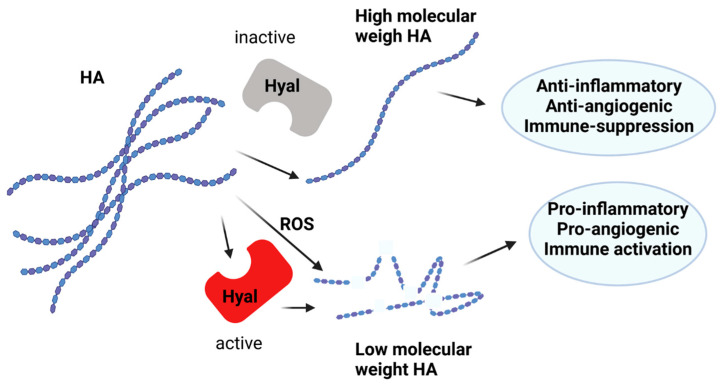
Inflammatory processes are controlled by HA of various molecular weights. An inactive hyaluronidase (Hyal) allows HMW HA to induce anti-inflammatory, anti-angiogenic, and immune-suppressive effects; an active Hyal cleaves HA to LMW HA fragments that inflict pro-inflammatory, pro-angiogenic, and immune activation effects.

Various reports emphasize that additional mechanisms to the TLR-dependent ones are involved in the chronic inflammation stage. Studies by Campo’s team proposed that HMW HA potentially physically masks TLR2 and TLR4 due to its large polymerized molecule size [104,105]. Currently, mechanisms that specifically regulate the influence of HA in acute and chronic inflammatory stages are still to be unveiled. The pro-and anti-inflammatory action of different sizes of HA molecules is summarized in Figure 4 and Table 1. However, additional factors, such as HA modifications, must be considered.

### 5.2. ROS and HA in Inflammation

ROS are essential in the immune response, but oxidative stress appears if there is an imbalance between the amount generated and the cell’s antioxidant capacity [106]. Thus, when ROS exceed the antioxidant capacity, they damage cellular proteins, lipids, and nucleic acids, mainly DNA [107]. The degradation of endogenous HA and additional modifications by ROS actions in producing active LMW HA fragments are well documented. Numerous studies show endogenous and exogenous HMW HA have a protective capacity against ROS.

Earlier studies in human corneal epithelial cells have demonstrated that UVB-induced oxidative damage can be reduced by exogenous HMW HA [108]. In addition, later studies have shown that in dry eye disease, the administration of HMW HA reduces the oxidative stress processes in the conjunctiva of patients [109].

In skin subjected to UVB irradiation, where LMW HA is intensively produced, inflammatory processes are attenuated upon applying extracellular superoxide dismutase (SOD). In this study, the complex inflammatory response was inhibited by SOD3 administration accompanied by suppression of the major histocompatibility complex II (MHCII), CD80, and CD86 expression in dendritic cells. The process was TLR4-dependent as the response could not be identified in TLR4-deficient mice. The inflammatory process was accompanied by NAPDH oxidase-dependent ROS generation, which could be blocked by the antioxidant effect of SOD3 [110]. The study by Kwon et al. describes a vicious cycle where UV damage stimulates HA production, ROS generation, and HA degradation into LMW HA, which in turn, through TLR4 receptors, augments the inflammation. Breaking the cycle, e.g., by attenuating ROS production or downregulating TLR4 expression, inhibits inflammation [110].

Exposure of human keratinocytes to contact sensitizers 2,4-dinitrochlorobenzene (DNCB) and PPD generates LMW HA fragments due to increased HYAL expression and ROS generation. In this model system, the generated LMW HA acts as a DAMP, stimulating the production of contact allergen-marker IL-18 [48]. Interestingly, LMW HA was shown to enhance HAS 1 and 2, TLR4 expressions, and downstream NF-κB signaling. TLR4 activation increases HAS expression and HA activity [111]. When keratinocytes were treated with an inhibitor of NADPH oxidase, downregulating ROS generation and subsequent HA degradation, their sensitization was partially blocked [48]. Other studies have shown that pro-inflammatory cytokines upregulate the expression of HAS genes, whereas inhibiting NF-κB attenuates this effect [112,113].

Recently, HAS3-dependent HA production was correlated with a protective effect against anthracycline-induced cardiac damage. The protective effects were attributed to the ROS-scavenging properties of HA [114].

The mechanisms of HMW HA hindering oxidative stress still need to be wholly understood, and several hypotheses have been generated. Thus, HMW HA has hydroxyl functional groups that are able to absorb ROS physically. Secondly, HMW HA interacts with CD44, probably activating pathways that dampen oxidative stress [115]. Thirdly, HMW HA can coat the cellular membrane and protect it from the direct action of ROS. Interestingly, the internalization of HA through CD44 receptor binding is suggested to neutralize intracellular ROS [116].

As previously stated, HMW HA reduces inflammation in osteoarthritis patients, where additional mechanisms, including its lubrication properties and antioxidant properties, are involved [117]. After intra-articular injection into the synovial fluid of patients, the ROS levels are reduced. Harvested human osteoarthritis chondrocytes prove high expression of stress response levels (e.g., transaldolase, annexin A1, elongation factor 2) that are decreased when HA is in contact with the harvested cells [118]. Recent reports show that HA-based compounds with specific properties exerting therapeutical action on cartilage regeneration also hinder ROS production in the inflamed joint.

A hydrogel consisting of phenylboronic acid, HA, and poly(vinyl alcohol) protected grafted chondrocytes from ROS in an animal model of osteoarthritis [119]. In another animal model focused on acute gouty arthritis and hyperuricemia, N-butylated-HA exerted anti-inflammatory and antioxidative properties. The study underlines that the anti-inflammation properties of HA are linked to its antioxidative ones [120].

Despite its pro-inflammatory properties, LMW HA has also been postulated to exert an antioxidative capacity. Thus, antioxidant properties were shown for LMW HA against ROS and inhibition of lipid peroxidation [121], but as there is limited literature on this topic, the mechanisms by which LMW HA performs its antioxidant properties are still subject to research. The interactions of LMW HA/HMW HA and ROS in inflammation are summarized in Table 2.

## 6. HA, ROS and Cancer

Cancer cells are thought to induce oxidative stress in the tumor microenvironment, recruiting surrounding ECM components to transport the signals to the adjacent normal stromal cells [122,123,124]. ROS production and hydrogen peroxide species generated modulate stromal fibroblasts to cooperate with cancer cells [123,125] and cause local and systemic inflammation via the innate immune response (NF-jB) [123]. Activated stromal cells in cancer facilitate changes in the ECM microenvironment, enhancing cancer progression and metastasis. ROS have been proven to facilitate tumorigenesis through several mechanisms, including induction of DNA damage, inflammation, changes in immune responses, regulation of signaling pathways that modulate autophagy and apoptosis, angiogenesis, and drug resistance [124,126,127].

Oxidative stress effects on the cells depend on the concentration and duration of the exposure [128,129]. High levels of ROS induce apoptosis or necrosis; low levels of ROS can promote cell proliferation facilitating tumor development [124,130]. Oxidative DNA damage, through ROS action, can initiate tumor development [131]. Mitochondrial respiration is a major source of ROS in most mammalian cells [132,133,134]. ROS production in cancer cells can result from enhanced metabolic activities due to aberrant growth factors, cytokine signaling, and oncogene activation [135,136,137,138]. In addition, increased activity of ROS-producing enzymes such as NADPH oxidase, cyclooxygenases, and lipoxygenases is evident [138,139]. In the tumor microenvironment, macrophages and neutrophils also produce ROS due to NADPH oxidase activation [139,140]. Superoxide is increased in cancer tissues [141] and is dismutated into H_2_O_2_, either by the mitochondrial MnSOD or the cytosolic Cu/ZnSOD [142,143]. In summary, ROS/RNS aid tumor development by regulating cell motility, invasiveness, and metastasis.

Thus, experimental evidence on invasive melanoma and lung metastasis introduces the involvement of a c-Met downstream signaling pathway resulting in the generation of hydrogen peroxide [144]. ROS induce activation of the Snail factor, the initiator of the epithelial–mesenschymal transition (EMT). More importantly, ROS are suggested to cause an environmental burden affecting cells programmed to metastasize [124,145].

As far as HA is concerned, it is overexpressed in most solid human tumors and affects cancer cell functions [15]. HA can regulate the cell motility of tumor cells [146,147,148,149,150]. HA influences tissue characteristics [10] and, in cancer, is shown to aid tumor growth, modulate immune surveillance and protect cancer cells from apoptosis [70]. Indeed, the HA receptor, CD44, is a major cell adhesion receptor expressed in cancer and cancer stem cells, which facilitates cell–cell and cell–matrix interactions, proliferation, differentiation, invasion, and migration [151,152].

HA’s action in cancer cell function is dependent on its molecular weight. LMW HA (1.2–500 kDa) produced during inflammation or tissue damage can lead to angiogenesis leading to cancer development [69,124,153,154,155,156]. HA fragmentation can either result from Hyal or ROS action [155,157] and can cause damage to the endothelial glycocalyx that results in endothelial barrier dysfunction, an initiating factor in disease states including cancer [77]. Moreover, HYBID was determined to facilitate colon cancer metastasis to the liver by regulating the infiltration of neutrophils [158], whereas, in breast and laryngeal cancer, HYBID expression is suggested to correlate with poor prognosis and has been proposed as a potential biomarker [159]. ROS were shown to increase the activity of HYALs by p38 MAPK regulation in lung inflammation [160]. Increased expression of HA in cancer, i.e., breast cancer induced by NOS, has been described [161]. HA enhances signaling affecting melanoma cell motility through CD44/EGFR interaction, and the FAK activation and ROS-dependent production of MMP-2 [148].

The interaction of tumor cells and monocytes in tissues involves surface molecules such as CD44, which are required to produce reactive oxygen intermediates (ROI). Experiments blocking CD44 on monocytes by HA addition diminish their reaction to tumor cells [162]. It was shown that HMW HA, and not LMW HA, enhances ROS levels in a manner dependent on CD44 [148,163]. HA/CD44 interaction produces peroxynitrite and H_2_O_2_, which induces HA-mediated melanoma cell motility. Experiments found that the above redox species are produced by NOX2 enzyme action [147], which triggers HA-induced PKC and Rac1 activity [148,164,165]. Upon HA binding to CD44, the expression of p62 is increased in breast cancer stem cells. P62, in turn, deactivates KEAP1 enhancing the activation of Nrf2, which triggers antioxidant response genes transcription. Finally, ROS accumulation is inhibited, and breast cancer drug resistance is enhanced [166].

CD44 exhibits various spliced isoforms, including CD44v [167], which combined with post-translational modifications, offer different cell function capabilities and signaling events in cancerogenesis dependent on or independent of HA [168]. Notably, several CD44 variants participate in reducing ROS levels in cancer cells by coupling with the glutamate-cystine transporter xCT [169]. The latter is identified as a mechanism of cell resistance to chemo- and radiotherapy [170]. It has been reported that CD44 v8-10 have a protective role in gastric cancer cells related to the redox stress-initiated Wnt signaling cascade and transcription of c-Myc [171]. CD44 is suggested to be a surface marker for cancer stem-like cells (CSCs), which are resistant to ROS-induced damage and have low levels of intracellular ROS [172]. It was shown that intracellular ROS generation after exposure to H_2_O_2_ was dependent on CD44v expression in different gastric and colorectal cancer cell lines [169]. CD44-regulated Hippo-signaling has been proven to sensitize glioblastoma cells to chemotherapy [173].

A recent study demonstrated that the inhibitor of the apoptosis-stimulating protein of p53 (iASPP) physically forms a complex with CD44s in normal and transformed cells. Various solid tumor types were found to exhibit discrete expression of iASPP, p53 and CD44s, whereas in fibroblasts, iASPP was shown to bind with CD44s via the ankyrin-binding domain in a manner stimulated by HA. Furthermore, a p53-induced increase in ROS production in HEK293 cells was dependent on CD44s expression. These authors suggest that the CD44, iASPP, and HA interaction may have therapeutical implications in transformed cells by regulating ROS generation [174].

Currently used cancer treatments and research for new treatments aim to increase the levels of ROS and RONS in cells to induce apoptosis [50,175,176]. On the same note, research in modulating the levels of HA and CD44 and their interactions is considered necessary to disrupt the signaling pathways that favor tumor progression and reduce cancer resistance to therapy.

## 7. Application of ROS–HA Interactions in Therapeutical Approaches

HMW and LMW HA in solid tumor microenvironments participate in tumor growth and progression, hinder anti-tumoral immune surveillance, and reduce cancer cell apoptosis [70]. In addition, CD44 HA receptor variants can couple with glutamate-cystine transporter xCT and reduce the intracellular ROS in cancer cells, making them resistant to chemo- and radiotherapy [177]. Therefore, the capability of HA receptors and HA to modulate ROS generation and levels must be considered when designing ROS-related therapies.

Notably, CD44 is highly expressed in tumor cells, and its binding to HA triggers downstream pro-tumoral signaling cascades. CD44 overexpression and its strong affinity to HA was recently explored in cancer targeting by developing nanoparticles that contain anti-tumoral drugs into which HA is incorporated. Thus, the HA-coated nanoparticles can target cancer stem cells by binding to activated CD44 and delivering drugs [178]. For example, HA nanoparticles delivered paclitaxel to B16F10 melanoma stem-like cells, inhibiting tumor sphere formation and downregulating cancer stem cells correlated transcription factor Oct-4 [179]. In addition, several recent studies have demonstrated that HA-based nanoparticles can attenuate tumor cell resistance to standard therapies.

Thus, in novel chemo-photothermal therapy, an ROS-responsive HA-based nanoparticle carrying methotrexate and a photothermal agent was designed. The administration of the respective nanomedicine inhibited almost 71% of tumor growth in a 4T1 tumor-bearing mice model. This recent study underlined the potential of ROS-responsive therapy in cancer [180].

Radio resistance is another important topic in oncology. Indeed, the up-regulation of NADPH oxidases in cancer cells leads to their adaptation to high ROS levels and hence limited sensitivity to radiotherapy. Therefore, nanoparticles incorporating HA to target CD44 and carrying an NADPH oxidases inhibitor were designed. These HA-based nanoparticles inhibited the tumor growth of breast cancer patient-derived xenografts in nude mice. Notably, topical low-dose radiotherapy was significantly more efficient in treated animals, demonstrating the increased radio sensitivity of cancer cells [176].

Nanoparticles utilizing HA as a carrier and incorporating a ROS generator (cinnamaldehyde) and β-phenethyl isothiocyanate to target tumor cells were generated differently. The designed nanoparticles inflict oxidative stress and suppress the activity of tumor cells’ antioxidant enzymes. In vitro and in vivo findings demonstrated the high antitumor efficacy of this HA-based nanoparticle [181].

Another recent report has shown that complex HA–ZnO nanoparticles delivering glucose oxidase and artesunate, compounds that induce cell starvation and oxidative damage, respectively, induced efficient tumor cell apoptosis and growth inhibition in a breast cancer BALB/c mice model [182].

Additionally, the disruption of specific HA and CD44 isoforms linkage could have a therapeutic effect on cancer. There are several methods to achieve this goal. For example, HA can be modified by HYALS that are chemically altered by deacetylation, sulfation, and oxidation [183], or even disrupted by gamma radiation [184]. In addition, as previously noted, ROS and RONs [e.g., hydroxyl (HO^•^) radicals and peroxynitrite (ONOO^−^)] can break the glycosidic bonds of HA [13].

Small interfering RNA or blocking antibodies can also disrupt CD44–HA interaction, exhibiting antitumor potency in animal models and even preventing tumor recurrence [185]. All these modifications hinder the CD44–HA bond and block the signaling events involved in the biology of tumor cells, from survival to metastasis.

Altering the CD44 structure is another possibility to disrupt the CD44–HA binding, as earlier shown by the reduction of disulfide bonds in the groove that binds HA [186]. More recently, it has been demonstrated that the oxidation of both players, HA and CD44, can efficiently disturb their interaction. The reduced binding downregulated downstream signaling events and attenuated the proliferation in cancer cells [50].

Various studies have highlighted the potential ROS-scavenging HA effect in therapeutical application. Thus, when coated with HA, bilirubin nanoparticles exhibited a superior ability to attenuate kidney damage due to ischemia compared with non-coated nanoparticles [187]. Likewise, it was shown that exogenous administration of HMW HA due to antioxidant properties is beneficial in treating lung diseases such as acute lung injury and airway hyperreactivity [77].

In another context, HA ROS scavenging abilities have been postulated to be beneficial as the standard therapies in oncology (e.g., radiotherapy chemotherapy, photodynamic therapy, chemo-photothermal therapy, etc.) increase the levels of ROS and RONS in cells to induce apoptosis [175]. HA is a powerful antioxidant that binds –OH groups, protecting cells from oxidative damage. When interacting with oxygen radicals, the HA molecule undergoes several changes, losing parts of its polymeric structure, which is also accompanied by fluid loss. Being a hygroscopic molecule with high osmotic properties, HA retains a large amount of water and ions, maintaining moisture and tissue turgor. Several studies indicate that this property of HA is important for controlling hydration and healing of ulcers of the oral mucosa [71,188].

Mucositis of the oral cavity is the most frequent and severe complication of conservative treatment of malignant tumors of the oropharyngeal zone, which significantly impacts the quality of life, the effectiveness of treatment of patients, and its cost [189,190,191].

Due to its properties and functions, HA can influence almost all the phases in the pathogenesis of radiation mucositis. In particular, by binding ROS, exogenous HMW HA inhibits the initiation phase of radiation mucositis, preventing oxidative damage to lipids, proteins, carbohydrates, and DNA in cells, thereby maintaining the viability of epitheliocytes and fibroblasts in the submucosa and blocking apoptosis [192,193,194,195]. The HMW HA molecule can attenuate the signal phase of mucositis since it binds to the CD44 receptor, inhibiting leukocyte recruitment and indirectly reducing the production of pro-inflammatory cytokines, which causes submucosal fibrosis and a decrease in the reparative capabilities of the mucosa [194,196]. The results of the studies also suggest that HA application results in accelerated ECM re-modeling due to the activation of fibroblasts that synthesize collagen. At the same time, as collagen deposition becomes more ordered, conditions are created for enhanced migration of epitheliocytes to the zone of mucosal damage [195,197,198]. Thus, HMW HA stimulates the healing phase.

The applications of ROS–HA interactions in therapeutical approaches are summarized in Table 3.

However, since exogenous HA can inhibit the action of ROS and suppresses oxidative stress, which is one of the critical links in the mechanism of the antitumor effect of radiation therapy, the safety of using this GAG in patients with malignant tumors requires additional studies.

## 8. Conclusions

ROS and RNS react with HA, producing LMW HA fragments that may bear various modifications. The generated LMW HA fragments modulate critical cellular signaling pathways that affect tissue homeostasis and participate in disease pathogenesis. The degradation of endothelial glycocalyx HA by increased ROS challenges vascular integrity and can initiate several disease progressions. On the other hand, HA exerts a vital role in wound healing through ROS-mediated HA modifications, which affect the innate immune system. Immune cells originating from innate and adaptive immunity express TLRs, which trigger the inflammatory processes upon LMW HA binding. CD44 is critical in transporting HA cues to the intracellular cascades regulating cell fate. Alternatively, ROS scavenging properties have been attributed to HA opening a novel field in ROS/RNS interactions. Therefore, the role of the longtime considered “archaic” HA molecule is more complex than presently perceived. ROS and RNS species interaction with HA may be a key to unlocking novel therapeutical options for human disease.

## Figures and Tables

**Table 1:** Different roles of LMWHA and HMWHA in inflammation events.

Type	Role	Reference
Low molecular weight hyaluronan	An HA tetra-saccharide is the smallest fragment to augment inflammation.	[89]
	An HA disaccharide (ΔHA2) blocks TLR4-dependent inflammation competitively.	[89]
	Activation of TLR4 on human dendritic cells (DC) through phosphorylation of p38/p42/44 MAP-kinases and nuclear translocation of nuclear factor (NF)-kappa B.	[71]
	An HA (15–40 kDa) isolated from obese patients induces, in normal PBMC, a significant increase in the expression of pro-inflammatory cytokines IL-1β, MCP-1, and IL-8.	[90]
	Increased deposition in bladder cancer enhances the production of multiple inflammatory cytokines, chemokines, and angiogenic factors.	[91]
	Stimulation of B cells via the TLR4 receptor increases secretion of IL-6 and TGF-beta.	[97]
	Enhancement interaction of CD44 with other receptors (TLR2, TLR4) and downregulation of CD44 association leads to NF-κB-specific transcriptional activation. Increases pro-inflammatory cytokine expression (IL-1β and IL-8) in tumor cells, including human breast cells.	[98]
High molecular weight hyaluronan	The increase in Foxp3 expression in the Treg subpopulation induces IL-2, IL-10, and TGF-β production and dampens inflammation.	[95]
	Increases macrophage recruitment in dendritic epidermal T cells (DETCs).	[96]
	Combination with platelet-rich plasma favors joint repair.	[101]
	Inhibition of IL-1β expression in human fibroblast-like synoviocytes from osteoarthritis patients.	[102,103]
	Its exogenous addition blocks CD44 and reduces pro-inflammatory cytokine gene expression.	[98]
	Physically masks TLR2 and TLR4 and modulates inflammation signaling.	[104,105]

**Table 2:** LMW HA/HMW HA and ROS in inflammation.

Type	Tissue/Cells	Action	Reference
Low molecular weight hyaluronan	UVB irradiated skin	NADPH oxidase-dependent ROS generation and HA degradation enhance inflammation through TLR4 receptors.	[110]
	Human keratinocyte cell line exposed to contact sensitizers	Increased HYAL expression and ROS generation result in HA fragmentation.	[48]
	Cyclophosphamide (CY) induced immunosuppressed mice	Antioxidant properties were shown for LMW HA against ROS and inhibition of lipid peroxidation.	[121]
High molecular weight hyaluronan	Human blood leukocytes	Internalization of HA through CD44 receptor binding neutralizes intracellular ROS.	[116]
	Osteoarthritis patients	After intra-articular injection of HA in the synovial fluid of patients, ROS levels are reduced.	[117]
	Human corneal epithelial cells	HA can reduce UVB-induced oxidative damage.	[108]
	Conjunctiva of patients with Sjogren’s syndrome	HA caused a reduction of oxidative stress processes.	[109]
High molecular weight hyaluronan complexes	Animal model of osteoarthritis	Phenylboronic acid, HMW HA, and poly(vinyl alcohol) complex protected grafted chondrocytes from ROS.	[119]
	Animal model of gouty arthritis	N-butylated-HA exerted anti-inflammatory and antioxidative properties.	[120]

**Table 3:** Use of HA in ROS-dependent therapeutical approaches.

Treatment Type	Mechanism	Reference
HA nanoparticles	Delivery of paclitaxel to B16F10 melanoma stem-like cells downregulated cancer stem cells (transcription factor Oct-4 correlation).	[179]
	ROS-responsive nanoparticles carrying methotrexate and a photothermal agent inhibited almost 71% of tumor growth in a 4T1 tumor-bearing mice model.	[180]
	CD44-targeted nanoparticles carrying an NADPH oxidases inhibitor in tumor growth in breast cancer.	[176]
	Nanoparticles with an ROS generator (cinnamaldehyde) and β-phenethyl isothiocyanate inflicted oxidative stress and suppressed the activity of tumor cells’ antioxidant enzymes.	[181]
	HA–ZnO nanoparticles delivering glucose oxidase and artesunate induced tumor cell apoptosis and growth inhibition in a breast cancer BALB/c mice model.	[182]
	HA–bilirubin nanoparticles exhibited a superior ability to attenuate kidney damage due to ischemia.	[187]
Hyaluronan-ROS scavenging abilities	Increased the levels of ROS and RONS in cells to induce apoptosis in oncology cases.	[175]
Disruption HA—CD44 interaction	HA modification by: (1) HYALS, deacetylation, sulfation, and oxidation (2) gamma radiation (3) ROS and RONs (e.g., hydroxyl(HO•) radicals, peroxynitrite (ONOO−)) can break the glycosidic bonds of HA.	(1) [183] (2) [184] (3) [13]
	Small interfering RNA or blocking antibodies exhibited antitumor potency in animal models.	[185]
	Changes in CD44 structure.	[186]
	Oxidation of both HA and CD44.	[50]
High molecular weight hyaluronan	Exogenous addition is indicated for the treatment of lung diseases such as acute lung injury and airway hyperreactivity correlated with ROS overproduction	[77]
	Exogenous addition inhibited the initiation phase of radiation mucositis, thereby preventing oxidative damage to lipids, proteins, carbohydrates, and DNA in cells, and maintaining the viability of epitheliocytes and fibroblasts in the submucosa and blocking apoptosis.	[192,193,194,195]
